# Editorial: Advanced Control Methods in Marine Robotics Applications

**DOI:** 10.3389/frobt.2021.654581

**Published:** 2021-04-15

**Authors:** Fabio Bonsignorio, Enrica Zereik, Marco Bibuli, Kristin Y. Pettersen, Oussama Khatib

**Affiliations:** ^1^ Heron Robots, Genoa, Italy; ^2^ Institute of Marine Engineering, Italian National Research Council, Genoa, Italy; ^3^ Department of Engineering Cybernetics, Centre for Autonomous Marine Operations and Systems, Norwegian University of Science and Technology, Trondheim, Norway; ^4^ Robotics Laboratory, Department of Computer Science, Stanford University, Stanford, CA, United States

**Keywords:** advanced control, control theory, control engineering, marine robotics, underwater robotics

## 1 Introduction

This article collection aims to provide a set of reports that could be used to better understand the state of the art and trigger a wide-range discussion among researchers and practitioners active in marine robotics and researchers active in robotics applications of control theory and engineering. This research topic originated from the workshop “Hand-Shaking Advanced Control in Marine Robotics Applications” held at IROS 2018. However, contributions have been collected through an open call.

As a result, we have a diverse set of contributions in which authors deal with current challenges in marine robotics; in particular issues and solutions related to control problems for systems interacting with the environment, advanced control of systems with uncertain dynamics working within uncertain environments, classical control with prioritized tasks for object avoidance, cooperative motion coordination, biomimetic approaches to formation control, etc. We did not receive submissions on more research-oriented topics like “embodiment,” “morphological computation,” “orchestration control,” “self-organization of behaviors in soft robots,” and “persistent autonomy,” nor on “human–robot interaction”; this is an interesting fact by itself.

## 2 Contributed Articles

The contributed articles provide a reasonably accurate state of the art for control methods currently used in marine robotics applications. Kelasidi et al. presents and integrates control for an underwater snake robot (this shape has a number of advantages in inspection and maintenance, the main issue being its control difficulty). Franchi et al. reports about the real-world experience and the technologies exploited during the European Robotics League in 2017. It is interesting to notice how “simple” the control techniques used in a real-world competition are with respect to other control approaches reported in the literature, while at the same time the team performed comparatively well. Garrido et al. shows (in simulation) the potential benefits of the fast marching method for navigation also in the marine robotics domain. Reis et al. addresses the cooperative moving path following (CMPF) control problem. It could be interesting to compare the three-layered hybrid collision avoidance (COLAV) system for autonomous surface vehicles in Eriksen et al. with that proposed in Kelasidi et al. Costanzi et al. reviews the interoperability issues in complex maritime activities performed by teams of heterogeneous robots. It is interesting here to compare with Berman et al., which merges a secure sampling method for environmental monitoring—based on distributed ledger technology—with a swarm management methodology inspired by Reynold’s boids. Could block-chain technologies help? Martinsen et al. reports about a reinforcement learning-based (RL) control scheme to optimize a model-based feedforward controller. De Palma et al. deals with the formation reconstruction for a team of vehicles based on the range distance between agents of a subset of the participants. Another interesting comparison is about swarm management in Berman et al. and cooperative control in Reis et al..

## 3 Conclusion

The diverse set of results published by a “real world–oriented” community probably gives a reasonable idea of where we are with robotics and AI at large.

Remarkably, a number of new tools are becoming available and are being exploited by the community. These include integrated guidance and control systems to ensure path following for underwater snake robots, new navigation methods, fast marching, new cooperative path following, obstacle and collision avoidance methods, new control approaches for range based navigation, reinforcement learning, and block-chain technologies among others. In particular, reinforcement learning seems a promising technique to improve the way robots perform most required tasks, while in general, deep learning is not widely used by the authors of this article collection. Fast marching holds promise for navigation tasks. Distributed ledger technologies seem to be ready for real-world application, to manage trustable and secure cooperation of heterogeneous multi-robot networks or swarms for critical applications (monitoring, but in general wherever security and trust are important).

Also in this community, a transition to a reproducible research practice is much needed, as only one contribution (Berman et al.) shared most of the code and data sets. The evaluation of the state of the art will certainly require further effort. However, marine robotics research is making inroads in this direction and increased progress can be expected in the coming years. Even from the down-to-earth point of view of marine robotics, we can expect quick and deep progress in the coming years.

